# Successful Management of an Occupational High-Voltage Electric Injury Associated With High-Risk Factors and a Clinically Significant Arrhythmia

**DOI:** 10.7759/cureus.41940

**Published:** 2023-07-15

**Authors:** Moturu Dharanindra, Ramesh Babu Pothineni, Dinesh Kumar Gontla, Srinivasa Rao P, Krishna Shriram Dhanasekaran

**Affiliations:** 1 Critical Care Medicine, Aster Ramesh Hospitals, Vijayawada, IND; 2 Cardiology, Aster Ramesh Hospitals, Vijayawada, IND; 3 Anesthesiology, Aster Ramesh Hospitals, Vijayawada, IND; 4 Research and Publications, Aster Ramesh Hospitals, Vijayawada, IND

**Keywords:** electrocution, arrythmia, ecg monitoring, electric burns, high voltage electric injury

## Abstract

Electric injuries are uncommon but can lead to numerous long-term complications as well as death. Occupational exposure is the most common cause of injury among the affected population. Both low-voltage and high-voltage electrocution are associated with significant morbidity and mortality. Patients with certain presentations are at a high risk of arrhythmia post-injury. Here, we discuss the presentation and management of high-voltage electrocution in a 35-year-old electrician.

## Introduction

Electric current refers to the movement of electrically charged particles. When individuals are exposed to a significant amount of electrical current, it can result in skin lesions, damage to internal organs, and even death. This type of injury is commonly referred to as electrocution [1]. Electrical injuries are typically categorized into low-voltage electric injuries (LVEI, <1000 volts), high-voltage electric injuries (HVEI, >1000 volts), and voltage not otherwise specified (NOS) [2,3]. Both low- and high-voltage injuries primarily occur in occupational settings, with individuals in their productive age range of around 30-40 years being most affected [2,4]. Occupational electrocution is more prevalent among men and ranks as the fourth leading cause of traumatic work-related fatalities [2,3]. In this report, we present a case study and discuss the management of a high-risk HVEI case resulting from occupational exposure.

## Case presentation

**Figure 1:**
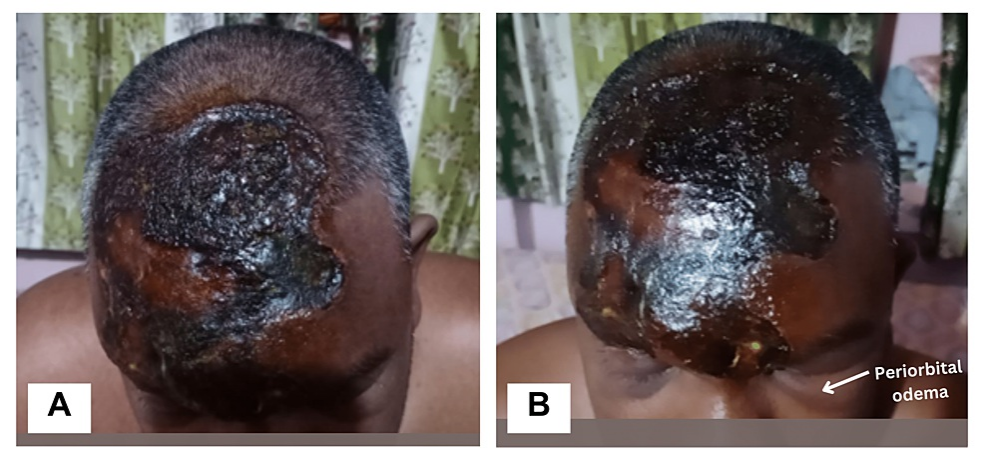
Entry site charred burn wounds in (A) bilateral supraorbital and (B) frontal regions associated with periorbital edema impairing eye openings.

A 35-year-old male electrician with no significant past medical history was brought to the Emergency Department (ED) immediately after an HVEI from a high-voltage battery. Following the electrocution, he suffered burns and multiple entry wounds on the right forehead. Upon arrival at the ED, the patient was in cardiac arrest, prompting the initiation of cardiopulmonary resuscitation (CPR) according to the Advanced Cardiac Life Support (ACLS) guidelines. CPR efforts were successful, resulting in a return of spontaneous circulation (ROSC). On further examination, third-degree burns (of body surface area, BSA < 9%) in the bilateral supraorbital and frontal regions associated with periorbital edema impairing eye-opening (i.e., large burn necrosis in both eyebrows and forehead) were noted (Figure 1A, 1B). Chemosis of the conjunctiva and sluggish pupillary response were also noted.

The patient was evaluated as per the Advanced Trauma Life Support (ATLS) guidelines. In a primary survey, he was found to be unstable with regard to respiration due to hypoxia (SpO_2_: 55%). The patient’s airway was secured with endotracheal intubation and ventilated. He was started with intravenous (IV) fluids, antibiotics, anti-tetanus prophylaxis, and topical silver sulphadiazine at the entry wound site. Upon admission, the Glasgow Coma Scale (GCS) score was E_1_ V_2_ M_2_. His blood pressure (BP) was 80/40 mmHg and his heart rate (HR) was 189 bpm (tachycardia). The blood glucose level was 143 mg/dL.

After ROSC, an arterial blood gas (ABG) analysis revealed compensated metabolic acidosis (pH 7.37; partial pressure of oxygen (PaO_2_), 62.3 mmol/L; partial pressure of carbon dioxide (PaCO_2_), 40.8 mmol/L; bicarbonate (HCO_3_), 10.4 mmol/L). It was corrected by ventilator adjustments and fluid resuscitation. The initial serum creatinine kinase level was 6234 IU/L, which was managed with IV fluids (IVF). It was subsequently monitored every six hours. The initial electrocardiogram (ECG) was suggestive of supra-ventricular tachyarrhythmia (SVT) with an HR of 195 bpm. It was successfully managed with synchronized electric cardioversion (50 J). The transthoracic echocardiogram (TTE) showed a good left ventricular (LV) function with a 65% ejection fraction (LVEF).

After an hour of ROSC, a computed tomography (CT) scan of the brain was done. The scan revealed a nodular calcification in the left frontal lobe, suggestive of a calcified granuloma. No evidence of intracranial injury was detected. A CT scan of the chest indicated sub-segmental atelectasis in the basal segments of the bilateral upper lobes, likely indicating aspiration pneumonitis. Additionally, collapse consolidations were observed in the posterior segments of the bilateral upper lobes, also consistent with aspiration pneumonitis. Minimal bilateral pleural effusion was present as well. A CT scan of the spine showed no internal injuries. The patient was closely observed and managed in the intensive care unit (ICU).

As the patient experienced symptomatic seizures, IV administration of levetiracetam and sodium valproate was initiated for seizure management. Frusemide was administered due to the presence of bilateral pleural effusion. Corticosteroids, such as dexamethasone and hydrocortisone, were given as supportive therapeutic agents. The necrotic burn areas were conservatively treated with IV n-acetyl cysteine (NAC) for a duration of two days, prednisolone eyedrops, and hydroxymethyl cellulose.

On postburn day two (PBD-2), a serial electroencephalogram (EEG) revealed bi-hemispheric dysfunction. Hypotension was observed and managed by administering vasopressors (IV noradrenaline) along with IV fluids (balanced crystalloids - Ringer's Lactate and Plasmalyte). The dosage of vasopressors was gradually reduced and eventually stopped. The patient's clinical condition improved, as evidenced by an improvement in GCS scores, and on PBD-3, the patient was successfully extubated without any complications. On PBD-4, a neurologic examination revealed that the patient was conscious, coherent, and oriented, obeying verbal commands. A sleep EEG performed at this time showed non-rapid eye movement (NREM) sleep in stages I and II. A repeat CT scan of the brain revealed soft tissue contusions in the bilateral frontal regions. An ophthalmic examination indicated conjunctival congestion, but the cornea and retina were within normal limits (WNL), and there were no complaints of disc edema.

**Figure 2:**
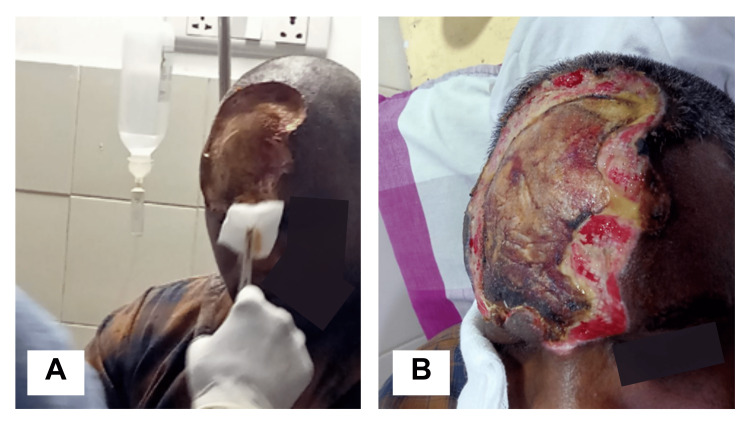
(A) Dressing post-debridement of the burnt site wound with surgical transection and repositioning. (B) Satisfactory healing of the burn wound with scarring after six days.

The burns were surgically debrided on PBD-2 and treated with silver sulfadiazine cream topically along with IV NAC and corticosteroids. Surgical dressing was performed post-debridement (Figure 2A). The patient’s condition improved with satisfactory healing of the burnt site (Figure 2B). Discharge was planned on PBD-6.

IV steroids were tapered and stopped. The patient was advised for follow-up and given oral anti-epileptics (levetiracetam and sodium valproate) and corticosteroids (oral - dexamethasone and ophthalmic prednisolone), bronchodilators (acebrophylline and budesonide + formoterol). Ophthalmic lubricants such as hydroxypropylmethylcellulose (HPMC) gel and polyethylene glycol (PEG) solution were also advised.

## Discussion

Electrical injuries comprise less than 5% of all hospital admissions worldwide; however, they are associated with significant morbidity and mortality. The average age of all electrical injury patients is 30.9 years, with 93.9% men and 6.1% women. The mode of injury in 75% of cases is occupational exposure [3,5,6]. In our case, the patient’s age was 35 years and he sustained HVEI due to occupational exposure. The presence of extensive burns (often seen in cases with HVEI), the extent of muscle and myocardial necrosis, the impact on the central nervous system, and the subsequent development of multiorgan dysfunction determine morbidity and long-term prognosis of patients [4,5].

HVEI diagnosis might be difficult without history and circumstantial evidence, as pathognomic features like electric marks and Joule burns are often seen only with low- or medium-voltage current involvement. Also, both entry and exit marks are seen together only in 20% of cases of HVEI [7]. HVEI causes variable degrees of cutaneous burns at the entry, exit, and hidden/deeper structures and serious cardiac derangements. Therefore, they should be treated in the ICU to monitor cardiac changes, hemodynamic conditions, and hidden injuries, such as muscle necrosis, after injury [6]. The patient was resuscitated with IV fluids and thorough cardiac monitoring during the first 24 hours. Mechanical ventilation (MV) was provided to obtain a normal oxygen saturation (SpO_2_: 99%).

It is crucial to differentiate between HVEI and LVEI due to the varying risks and occurrence of delayed arrhythmias. Several studies have shown that delayed arrhythmias are associated with HVEI but not with LVEI [8-11]. Considering patient safety, both the ATLS and European Resuscitation Council (ERC) guidelines mandate inpatient monitoring for life-threatening cardiac rhythm disturbances in all HVEI cases [11]. In our case, the patient's ECG was continuously monitored in the ICU. Initially, the patient exhibited sinus tachycardia, but no delayed arrhythmias or significant ECG changes were observed during the hospital stay and follow-up period.

Some investigators suggest certain high-risk factors for post-HVEI arrhythmia [11-13]. High-risk factors in our patient were high voltage, a transthoracic pathway of the electric current, tetanic muscular contractions, loss of consciousness, cardiac arrest, ECG changes, and rhythm abnormality on presentation. Although a clinically significant arrhythmia (sinus ventricular tachycardia) was detected in baseline ECG, it was managed accordingly. There was no presentation of delayed arrhythmias or ECG changes on PBD-2 to PBD-6 and during the follow-up.

Overall, the management of HVEI requires a multidisciplinary approach involving resuscitation, monitoring, supportive care, and treatment of specific complications associated with the injury.

## Conclusions

This case serves as a reminder of the significant morbidity and mortality associated with electrical injuries, particularly HVEI. It highlights the importance of early recognition, prompt resuscitation, and comprehensive management to improve patient outcomes. Further research and studies are needed to enhance our understanding of the pathophysiology and optimal management strategies for HVEI, ultimately leading to improved patient care and outcomes in the future.
